# Prevention and management of kinesiophobia after total knee arthroplasty: an evidence-informed narrative review and proposed stepped-care pathway

**DOI:** 10.3389/fresc.2026.1962328

**Published:** 2026-10-19

**Authors:** Lina Liu, Haobo Sun, Li Ni

**Affiliations:** 1 Tongji University School of Medicine, Shanghai, China; 2 Department of Orthopedics, Naval Medical Center of PLA, Shanghai, China; 3 The East Hospital Affiliated to Tongji University, Shanghai, China

**Keywords:** cognitive behavioral therapy, fear of movement, kinesiophobia, patient education, rehabilitation, stepped care, Tampa scale for kinesiophobia, total knee arthroplasty

## Abstract

Kinesiophobia is a potentially modifiable biopsychosocial barrier to recovery after total knee arthroplasty (TKA), yet evidence specific to fear of movement is dispersed across rehabilitation, psychological, nursing, and perioperative literature and is often conflated with evidence for general TKA rehabilitation. This evidence-informed narrative review aimed to synthesize current evidence for the assessment, prevention-oriented management, and treatment of kinesiophobia across the TKA pathway while distinguishing fear-specific findings from broader rehabilitation evidence. PubMed/MEDLINE and the Physiotherapy Evidence Database (PEDro) were searched from inception through 15 September 2026; the Cochrane Library, current rehabilitation guidance, backward and forward citation tracking, and bibliographic verification were also used. Evidence was interpreted according to study design, directness to kinesiophobia outcomes, consistency, and reported limitations. Available studies indicate that kinesiophobia is associated with pain, pain catastrophizing, lower self-efficacy, impaired physical performance, and poorer participation after TKA, although causal relationships remain uncertain. Direct TKA studies have reported reductions in kinesiophobia with multicomponent fear-focused functional exercise, selected educational or cognitive-behavioral approaches, and some technology-supported interventions; however, effects on pain and physical function are inconsistent, and many trials are small or single-center. Multimodal analgesia, early supported mobilization, progressive strengthening, and telerehabilitation remain important components of TKA rehabilitation, but their general effectiveness should not be interpreted as evidence of a specific antikinesiophobia effect unless fear outcomes were assessed. Tampa Scale for Kinesiophobia measures can support repeated multidomain assessment, but thresholds and clinically important changes are not interchangeable across versions, languages, populations, or postoperative time points. On this basis, we outline a clinically oriented screening schedule and a stepped-care pathway that prioritize exclusion of medical or surgical barriers, realistic education, supported movement, fear-focused functional practice, self-efficacy enhancement, and selective psychological or digital adjuncts. These frameworks are intended for local adaptation and prospective validation. More rigorous multicenter studies are needed to establish clinically meaningful change in kinesiophobia and to determine which patients benefit from targeted interventions, as well as optimal dose, durability, safety, and cost-effectiveness.

## 1 Introduction

Total knee arthroplasty (TKA) is an effective treatment for end-stage knee osteoarthritis, but recovery is heterogeneous. Global osteoarthritis burden continues to rise, and a substantial minority of patients experience persistent pain, delayed functional recovery, or dissatisfaction despite technically successful surgery (1–3). A purely biomedical explanation is insufficient for this variability because beliefs, expectations, affective distress, pain appraisal, social support, and confidence in movement influence engagement with rehabilitation.

Kinesiophobia is commonly defined as excessive or disabling fear of movement arising from a perceived vulnerability to pain, harm, or reinjury. In the TKA setting, the feared behavior—loading, flexing, walking, stair use, or exercising—is also central to recovery. A recent systematic review and meta-analysis estimated a pooled postoperative prevalence of approximately 35%, although estimates varied by timing, population, threshold, and study quality (4). Earlier synthesis linked higher kinesiophobia with worse pain and functional outcomes for up to 1 year after knee replacement (5). Contemporary studies identify interacting correlates rather than a single causal determinant: greater pain, lower quadriceps strength, poorer sit-to-stand performance, pain catastrophizing, low rehabilitation self-efficacy, psychological distress, limited social support, and socioeconomic or educational constraints have all been associated with higher fear of movement (4, 6–16). Trajectory research also suggests that patients do not follow a single recovery pattern; some improve rapidly whereas others maintain or develop high fear with poorer rehabilitation outcomes (7).

The clinical challenge is that evidence directly targeting kinesiophobia after TKA is mixed with a much larger literature on general arthroplasty rehabilitation. Interventions such as adequate analgesia, early mobilization, progressive strengthening, and telerehabilitation may be appropriate components of standard care, but their general effectiveness does not by itself demonstrate a fear-specific treatment effect. This review therefore distinguishes direct evidence—studies in TKA that measured kinesiophobia or closely related movement fear—from indirect or contextual evidence supporting general rehabilitation principles.

The purpose of this review is to synthesize the most relevant current evidence for identification, prevention, and management of kinesiophobia after primary TKA, while making the limits of the evidence explicit. We address whom to assess, how TSK scores should be interpreted, which interventions have direct fear-related evidence, which components are supported primarily by general TKA rehabilitation evidence, and how these elements could be organized into a proposed stepped-care pathway for prospective evaluation.

## 2 Methods for this evidence-informed narrative review

### 2.1 Review design and clinical question

This article was designed as an evidence-informed narrative review rather than a *de novo* systematic review. The clinical question was framed using a population-concept-context approach: adults undergoing primary TKA; prevention, identification, and management of kinesiophobia or closely related fear-avoidance behavior; and preoperative, inpatient, outpatient, home, and digital rehabilitation settings. The review was intended to integrate clinically relevant evidence across disciplines while explicitly distinguishing studies that measured fear-related outcomes from evidence supporting rehabilitation more generally.

### 2.2 Search sources, dates, and strategies

Literature searches were conducted from database inception through 15 September 2026. PubMed/MEDLINE and the Physiotherapy Evidence Database (PEDro) were searched. The Cochrane Library was searched for relevant systematic reviews, and guideline-source searching targeted current physical-therapy guidance for TKA and Enhanced Recovery After Surgery (ERAS) perioperative recommendations. Backward citation tracking from included systematic reviews and key trials and forward citation checking of sentinel studies were used to identify additional direct TKA evidence. Bibliographic metadata for all references retained in the final manuscript were subsequently cross-checked against PubMed and/or publisher records.

The core PubMed/MEDLINE concept strategy was: (kinesiophobia OR “fear of movement” OR “fear avoidance” OR “Tampa Scale” OR catastrophiz*) AND (“total knee arthroplasty” OR “total knee replacement”). Supplementary searches paired TKA with cognitive behavioral therapy, pain neuroscience education, preoperative education, graded exposure, first mobilization, early mobilization, exercise, strengthening, motor imagery, virtual reality, telerehabilitation, mobile health, self-efficacy, psychological predictors, and chronic postsurgical pain. PEDro searches used the combinations “kinesiophobia total knee arthroplasty”, “fear movement total knee arthroplasty”, “cognitive behavioral total knee arthroplasty”, and “virtual reality total knee arthroplasty”. The Cochrane Library was checked using the core kinesiophobia/fear and TKA concepts and the principal intervention terms.

Embase, CINAHL, PsycINFO, Scopus, and Web of Science were not independently searched. To broaden disciplinary coverage within the scope of a narrative review, relevant recent systematic reviews that had searched rehabilitation, nursing, psychological, and biomedical databases beyond MEDLINE were retained (17–19), and direct TKA studies identified through PEDro and citation tracking were added. The absence of independent searches of these databases remains a limitation; therefore, the review should not be interpreted as an exhaustive systematic retrieval of all eligible literature.

### 2.3 Eligibility and evidence prioritization

Priority was given to systematic reviews/meta-analyses, clinical practice guidelines, randomized controlled trials, and prospective cohort studies. Evidence was classified as direct when participants underwent TKA and kinesiophobia, fear of movement, fear of mobilization, or a closely related fear-avoidance outcome was measured. Evidence was classified as partly direct when a body of evidence combined fear-specific TKA studies with broader TKA rehabilitation studies. General TKA rehabilitation evidence without a fear-related outcome and evidence from other musculoskeletal pain populations were treated as indirect or contextual. Cross-sectional and qualitative studies were retained when they clarified potentially modifiable factors, patient experience, or implementation needs. Case reports, nonclinical opinion pieces, unverified preprints, and studies without a fear-related or rehabilitation-relevant outcome were not used as the principal basis for clinical statements.

### 2.4 Appraisal and formulation of clinical statements

Because this was a narrative review, no *de novo* study-level risk-of-bias assessment or formal GRADE or Joanna Briggs certainty framework was applied. Clinical statements were therefore not assigned formal recommendation-strength grades. Evidence was interpreted narratively according to study design, directness to TKA-related kinesiophobia, consistency, sample size, duration of follow-up, and limitations reported by the primary studies, systematic reviews, or guidelines. Where a systematic review or guideline reported risk-of-bias or certainty judgments, those assessments informed the narrative interpretation but were not converted into a new grade.

Table 1 presents evidence-informed clinical statements rather than graded recommendations. Each statement is accompanied by an explicit directness label (direct, partly direct, indirect/contextual, or consensus/safety) and a brief description of limitations. Wording was deliberately calibrated to the evidence: “supports” or “suggests” is used for consistent direct evidence; “may be considered” for uncertain or small-study evidence; and “is reasonable as part of standard TKA care” for contextual rehabilitation practices that are not themselves established treatments for kinesiophobia.

**Table 1:** Evidence-informed clinical statements.

Clinical statement	Directness to TKA kinesiophobia	Evidence basis	Interpretation/limitations
Repeated multidomain assessment rather than a stand-alone TSK score is reasonable when fear appears to affect recovery.	Direct	Systematic/observational TKA evidence on prevalence, trajectories, correlates, adherence, and participation (4–12).	Direct association evidence supports assessment, but no trial validates the proposed screening intervals or a universal referral threshold.
Use a validated language-specific TSK version and interpret scores with item content, observed behavior, pain, catastrophizing, mood, self-efficacy, and function.	Partly direct	TKA observational evidence plus cross-condition TSK psychometric reviews (7–10, 18, 19).	Thresholds and meaningful change are not interchangeable across TSK versions, languages, or postoperative time points; TSK-17 ≥ 37 is not a universal diagnostic cutoff.
Exclude medical, surgical, neurological, hemodynamic, and analgesic barriers before attributing delayed mobilization to kinesiophobia.	Consensus/safety	Perioperative rehabilitation guidance and clinical safety principles (28, 29).	Necessary for safe care, but not tested as a kinesiophobia intervention.
Structured preoperative education can be used to address fear, expectations, first mobilization, red flags, and self-management.	Direct	Recent TKA education studies including randomized trials and a single-group pre-post study (23–25).	Interventions, populations, and outcomes differ; the single-group study cannot isolate treatment effect.
Multimodal analgesia can support participation in rehabilitation but should not be presented as a stand-alone treatment for kinesiophobia.	Indirect/contextual	Enhanced recovery and TKA guidance (27–29).	Strong rationale for function and recovery; fear-specific effects generally not measured.
Early supported mobilization is appropriate once physiologically stable; fear related to first mobilization can be addressed with preparation and support.	Partly direct	General early-mobilization evidence and guidance plus a randomized first-mobilization protocol assessing fear-related outcomes (27, 29, 30, 32).	Fear of first mobilization and persistent kinesiophobia are related but not equivalent constructs.
For activity-specific fear, fear-focused functional exercise and a collaborative graded task hierarchy may be incorporated within rehabilitation.	Direct+indirect	TKA RCT of functional exercise aimed at managing kinesiophobia, small exercise trial, and broader chronic-pain exposure literature (33–35).	Direct TKA evidence is multicomponent; isolated graded exposure has not been robustly tested after TKA.
Progressive strengthening, gait, balance, and task-specific practice should remain part of standard TKA rehabilitation; when fear is present, pair them with explanation, feedback, and mastery.	Partly direct	General TKA rehabilitation reviews/guidelines plus selected fear-outcome studies (21, 29, 31, 34, 44).	General rehabilitation benefit should not be misrepresented as direct kinesiophobia efficacy.
Target CBT-informed care to persistent fear, catastrophizing, distress, repeated avoidance, or rehabilitation plateau rather than promising universal benefit.	Direct	TKA meta-analyses and mixed RCTs (17, 36–41); broader kinesiophobia CBT meta-analysis is indirect (42).	Kinesiophobia may improve, but effects on pain and knee function are inconsistent and interventions are heterogeneous.
Self-efficacy support, credible communication, and family coaching are reasonable adjuncts when they address identified barriers.	Direct observational/qualitative	Mediation, network, social-support, and qualitative TKA studies (4, 8, 9, 12, 20, 43).	Mechanistic and association evidence is clinically relevant, but isolated intervention effects are not established.
Digital education, mobile health, VR, motor imagery, or motivational procedures may be considered as adjuncts when they improve access, engagement, feedback, or exposure.	Direct but heterogeneous	Direct TKA trials for video, mobile health, VR, motor imagery, and motivational procedures plus broader telerehabilitation/XR evidence (21, 45–53, 55).	Many studies are small, single-center, short-term, or use different intervention components; long-term and comparative effectiveness remain uncertain.
Passive complementary modalities may be used only as optional adjuncts when safe and when they facilitate, rather than replace, active rehabilitation.	Direct but limited	Small randomized reflexology study and relaxation-related trial (49, 54).	Evidence is limited; avoid reinforcing beliefs that movement is unsafe without passive treatment.
Persistent or worsening fear with disproportionate pain, distress, red flags, or marked functional loss warrants coordinated reassessment.	Consensus/partly direct	Trajectory/observational evidence, pain literature, and safety reasoning (7, 10, 16, 22).	Escalation criteria require local pathways; no validated universal TSK threshold defines multidisciplinary referral.

These statements are descriptive clinical interpretations rather than formally graded recommendations. Directness labels indicate whether the cited evidence measured kinesiophobia or closely related movement fear after TKA; they do not represent a formal certainty-of-evidence rating.

### 2.5 Bibliographic verification

On 19 September 2026, every reference retained in the final manuscript was cross-checked against PubMed and, when needed, the publisher record. Author order, exact article title, journal, year, volume, pagination or article number, DOI, and publication status were reconciled. Articles available online without final volume/page assignment were identified as in press. No unverified preprints or unpublished reports were used to support the clinical statements.

## 3 Clinical meaning and assessment

### 3.1 Kinesiophobia is a dynamic rehabilitation barrier

Fear after TKA is not inherently pathological. Transient caution can be adaptive when it prompts safe use of walking aids and adherence to precautions. Kinesiophobia becomes clinically relevant when threat beliefs appear disproportionate to the current clinical situation, persist despite appropriate reassurance and medical stability, or drive avoidance that interferes with mobility, range-of-motion work, strengthening, sleep, self-care, or social participation. Qualitative evidence indicates that patients describe overcoming fear through informed movement, trusted support, and hope, themes that are consistent with education, supervised mastery, and recovery-focused communication (20).

Kinesiophobia should not be conflated with pain intensity, anxiety, depression, pain catastrophizing, fear of falling, or low self-efficacy. These constructs overlap but are not interchangeable. Network and mediation studies suggest that rumination and coping self-efficacy may act as bridge variables linking catastrophizing, fear, and adherence (8, 9). A multidomain assessment is therefore more clinically informative than a TSK score alone.

### 3.2 TSK interpretation, thresholds, and change scores

The Tampa Scale for Kinesiophobia exists in several versions, including the TSK-17, TSK-13, and TSK-11. A 2023 systematic review of musculoskeletal populations found generally acceptable reliability and responsiveness for several versions but noted inconsistent validity for the TSK-11 and favored the TSK-17 or TSK-13 when broadly applicable psychometric support is required (18). A 2024 COSMIN-informed review of cross-culturally adapted TSK-11 versions identified 15 languages and substantial heterogeneity in measurement properties, with concerns about measurement error and limited certainty for responsiveness (19). These findings are not specific to TKA, but they caution against transferring one threshold or change score across versions or cultures.

Some TKA studies have used a TSK-17 score of 37 or greater to identify a high-fear subgroup, including a recent motor-imagery trial (21). That value should be interpreted as a study-specific enrollment or decision threshold, not as a universal diagnostic cutoff. Likewise, the TSK-11 does not have a universally validated threshold for all TKA populations and postoperative time points. Clinicians and researchers should use a validated language-specific version, report the version and scoring method, interpret scores together with item content and observed behavior, and avoid converting thresholds or change values between TSK versions. A clinically important change specific to TKA, language, and recovery phase remains insufficiently established.

### 3.3 Proposed screening schedule

Table 2 presents a proposed, non-validated screening schedule intended to structure clinical assessment and local implementation. The intervals are pragmatic rather than evidence-derived, and they should be adapted to the local pathway, validated instruments, length of stay, and patient needs. The schedule should not be interpreted as a validated diagnostic or triage algorithm.

**Table 2:** Proposed screening schedule for local adaptation. This schedule is not a validated clinical algorithm. TSK, Tampa Scale for Kinesiophobia. Use a validated language-specific version where available; scores are decision aids rather than stand-alone diagnoses.

Time point	Minimum assessment	Clinical purpose
Preoperative assessment (approximately 2–6 weeks when feasible)	Validated local TSK version or brief fear screen; pain; catastrophizing; anxiety/depression; self-efficacy; baseline transfer/gait function	Identify beliefs or behaviors that may require tailored education, supervised practice, pain planning, or referral. Do not use one TSK cutoff as a diagnosis.
Postoperative day 0–1 or before first mobilization	Brief movement/mobilization fear rating; pain at rest and movement; orthostatic symptoms; motor/sensory status; mobilization readiness	Differentiate fear from physiological instability, neurological block effects, uncontrolled pain, or surgical/medical barriers; prepare supported first mobilization.
Discharge/transition to home	Observed transfers/walking/stairs; confidence with home plan; red flags; repeat brief fear assessment or TSK when clinically useful	Identify avoidance that may interfere with the home program and agree on achievable functional goals.
Early outpatient/home follow-up (commonly 2–6 weeks)	Fear/TSK trajectory; adherence; pain interference; swelling/sleep; self-efficacy; range of motion; strength; walking and patient-valued activity	Identify persistent or worsening fear and decide whether enhanced rehabilitation or CBT-informed input is warranted.
Around 3 months and whenever recovery plateaus	Repeat multidomain assessment; screen for surgical, prosthetic, neuropathic, chronic pain, mood, or social contributors	Avoid diagnostic overshadowing; escalate medical, pain, rehabilitation, or psychological review when indicated.

### 3.4 Interpretation and differential diagnosis

TSK responses should be interpreted with item-level beliefs and observed function. Endorsement of beliefs that movement is dangerous or that the body is seriously damaged may identify a target for pain and safety education, whereas avoidance driven by dizziness, wound concerns, severe swelling, extensor weakness, or uncontrolled pain requires medical or rehabilitation management first (9, 10).

Before escalating psychological treatment, clinicians should assess for infection, venous thromboembolism, fracture, extensor mechanism dysfunction, instability, arthrofibrosis, neurological deficit, severe anemia or orthostatic intolerance, medication adverse effects, and uncontrolled or neuropathic pain. Persistent pain and fear can coexist with a correctable surgical or medical problem; a biopsychosocial approach must not become diagnostic overshadowing.

## 4 Evidence for prevention and early risk reduction

### 4.1 Preoperative risk stratification and expectation-setting

Preoperative assessment can identify patients who may need additional support. Relevant domains include prior difficult pain experiences, catastrophic expectations, avoidance patterns, self-efficacy, anxiety/depression, health literacy, social support, baseline mobility, and practical barriers to exercise. Psychological factors—particularly catastrophizing and anxiety—are associated with postoperative pain and recovery, but effect estimates are heterogeneous and should not be used deterministically (16, 22). A high fear score indicates a need for contextual assessment rather than an inevitable poor outcome.

There is direct but heterogeneous evidence that structured education can influence fear-related outcomes. A 2026 randomized trial of nurse-led preoperative education in older adults undergoing TKA reported lower kinesiophobia and improved mobilization indicators compared with standard education (23). A 2024 randomized study using health education based on the transtheoretical model reported lower kinesiophobia and improved selected rehabilitation outcomes (24). By contrast, a 2025 study of preoperative education and fear of first mobilization used a single-group pretest-posttest design, so causal inference is limited (25). These studies support education as a plausible component of care but do not establish one universal program, dose, or timing.

Education should explain the expected course of pain, swelling, bruising, stiffness, sleep disturbance, and temporary functional decline; distinguish expected discomfort from warning signs; demonstrate safe use of aids; rehearse the first mobilization and home exercises; and use teach-back. Functional milestones are preferable to promises of being pain-free.

### 4.2 Prehabilitation and mastery experiences

Prehabilitation may provide mastery experiences, familiarize patients with exercises, and permit supervised practice of feared tasks. However, broad prehabilitation programs do not consistently improve all postoperative outcomes, and a randomized trial found no clear superiority of a multidisciplinary prehabilitation program for functional independence after TKA (26). Importantly, most general prehabilitation studies did not select patients by kinesiophobia or test fear-specific mechanisms. Prehabilitation should therefore be presented as a general rehabilitation strategy with potential relevance to confidence and movement exposure, not as established prevention of kinesiophobia.

### 4.3 Analgesia and early mobilization as contextual enablers

Pain is repeatedly associated with kinesiophobia, but eliminating all pain before movement is neither realistic nor required. Multimodal analgesia is standard perioperative care because it can support sleep, participation, and progressive activity while minimizing adverse effects that impair mobilization (27–29). This evidence is primarily about TKA recovery and safety rather than a direct anti-kinesiophobia effect; analgesia should therefore be described as an enabler of rehabilitation rather than a stand-alone treatment for fear of movement.

Similarly, early supported mobilization is a central component of enhanced recovery and physical therapy pathways when patients are physiologically stable (27, 29–31). A recent randomized first-mobilization protocol improved readiness and mobilization time and reduced anxiety and several mobilization-related fears; although the Tampa Scale for Kinesiophobia was administered, the published abstract reports effects on readiness, mobilization time, anxiety, and specific fears rather than a between-group global TSK result (32). This provides direct evidence for selected first-mobilization fears but should not be interpreted as proof that early mobilization prevents persistent kinesiophobia.

## 5 Evidence for management

### 5.1 Fear-focused functional exercise and graded exposure

When fear is clearly linked to specific activities, repeated safe performance of feared and meaningful tasks can be integrated into rehabilitation using a collaborative hierarchy. The therapist can elicit the anticipated harm, select a tolerable task, monitor symptoms and fear, compare the predicted and actual response, reinforce successful movement, and advance difficulty as confidence and technique improve. This approach is clinically coherent, but the evidence after TKA should be described precisely.

A randomized trial of 110 patients reported that home-based functional exercises explicitly aimed at managing kinesiophobia improved disability, fear-avoidance, pain, and quality of life compared with advice to remain active, with benefits observed through 6 months (33). This is important direct evidence, but the intervention was multicomponent and does not isolate graded exposure as a single technique. A small randomized trial of elastic-band exercise also reported improvement in kinesiophobia and selected functional or psychological outcomes (34). Evidence for graded exposure as an isolated treatment largely comes from broader chronic pain literature (35). Accordingly, a function-led exposure approach may be incorporated for patients with activity-specific fear, but it should not be presented as a strongly established stand-alone TKA intervention.

### 5.2 Cognitive-behavioral and self-efficacy strategies

Cognitive-behavioral approaches target catastrophic interpretations, avoidance, unhelpful attention to symptoms, and low confidence. A 2024 TKA-specific meta-analysis found short-term reduction in kinesiophobia after cognitive-behavioral therapy (CBT), but no consistent improvement in pain or knee function (36). A 2026 meta-analysis suggested benefits across selected pain, function, psychological, or range-of-motion outcomes, but intervention content and timing were heterogeneous (37). Individual TKA trials are mixed: some CBT-informed programs improved fear-related outcomes, whereas other trials in older adults or high-catastrophizing patients did not consistently improve pain or function (38–41). A broader 2025 meta-analysis across diagnoses supports CBT for kinesiophobia but is indirect for TKA (42).

CBT-informed care is therefore better targeted to patients with persistent fear, prominent catastrophic beliefs, repeated avoidance despite medical stability, significant distress, or rehabilitation plateau rather than applied as a universal intensive intervention. Brief techniques such as collaborative goal setting, cognitive reframing, behavioral experiments, pacing, relaxation, and reinforcement of mastery may be incorporated within clinicians' scope of practice, while complex or severe presentations warrant referral to psychology or pain services. Self-efficacy can be supported through achievable goals, visible progress, specific feedback, patient choice, and successful movement experiences; however, much of the TKA evidence for self-efficacy as a mechanism remains observational (8, 9, 43).

### 5.3 Progressive exercise within standard TKA rehabilitation

Range-of-motion practice, quadriceps activation, progressive strengthening, balance, gait training, and task-specific endurance are supported components of TKA rehabilitation (29, 31, 44). Their inclusion in a kinesiophobia pathway is clinically appropriate because patients need physical capacity and repeated successful movement experiences. Nevertheless, most general rehabilitation trials and guidelines were not designed to test whether these components prevent or treat kinesiophobia. Direct fear-related evidence is available for selected interventions such as elastic-band exercise (34), motor imagery added to rehabilitation in a high-fear sample (21), and some early rehabilitation programs, but it is not appropriate to generalize those findings to every exercise modality. Exercise should therefore be paired with explanation, feedback, and functional goals when fear is present, while maintaining the distinction between general rehabilitation benefit and fear-specific efficacy.

### 5.4 Communication, social support, and participation

Communication can reduce or amplify threat. Credible, behavior-linked messages should acknowledge symptoms while explaining the current loading plan and progression. Teach-back can confirm understanding of expected symptoms, red flags, analgesic timing, walking-aid use, and the home program. Family members can be coached to support safe independence rather than inadvertently reinforce avoidance.

Low social support has been associated with greater kinesiophobia, while qualitative work highlights the importance of trusted assistance, information, and hope (4, 20). Higher kinesiophobia has also been associated with less favorable social participation profiles at 3 months (12). These findings justify assessing the social context, but they are observational or qualitative and do not establish the independent effectiveness of family coaching or communication scripts.

### 5.5 Digital, virtual, and imagery-based adjuncts

Digital interventions are increasingly relevant, but the directness of evidence varies. Micro-video education reduced fear and improved early recovery in one study conducted during pandemic restrictions (45). A 2026 randomized trial of a mobile education and exercise program beginning preoperatively and continuing after discharge reported lower kinesiophobia and better selected recovery outcomes than usual care (46). A separate 2026 randomized trial found that an AI-assisted motivational procedure delivered before mobilization reduced short-term TSK scores and improved mobility measures; the study was single-center and does not establish long-term effectiveness (47).

Virtual reality (VR) and imagery studies also provide direct but generally small-study evidence. A randomized trial of immersive VR plus exercise included only 21 women and reported improvements in kinesiophobia and selected functional outcomes (48). VR-based progressive relaxation reduced early postoperative pain and TSK scores in a 2026 randomized study (49). A 2025 randomized trial of motor imagery in 40 patients selected using TSK-17 ≥ 37 reported improvements in psychological and physical outcomes (21); the selection threshold should not be generalized as a universal cutoff. Broader extended-reality reviews show potential short-term benefits for pain, function, or anxiety but do not consistently use kinesiophobia as the primary outcome (50–52).

Telerehabilitation has evidence for general TKA rehabilitation outcomes and can improve access, monitoring, and continuity (53), but this should be considered contextual rather than direct evidence of a kinesiophobia effect unless fear outcomes are measured. Digital literacy, accessibility, privacy, and escalation pathways should be addressed, and technology should complement rather than replace individualized progressive loading and real-world task practice.

### 5.6 Passive complementary interventions

A double-blind randomized study reported that foot reflexology reduced pain and kinesiophobia after TKA (54). The finding is clinically interesting but derives from a small single-center sample. Passive modalities or relaxation can be considered as optional adjuncts when safe and preferred by the patient, particularly if they facilitate participation, but they should not displace active rehabilitation or be framed as necessary for safe movement.

## 6 Evidence-informed clinical statements

Table 1 summarizes clinical statements without formal grades. The directness column is intended to prevent indirect general rehabilitation evidence or consensus-based safety principles from being presented with the same certainty as direct TKA studies that measured fear-related outcomes.

## 7 Proposed stepped-care pathway

Table 3 is a proposed implementation framework, not a validated clinical algorithm. Its purpose is to organize escalation from universal rehabilitation to more targeted psychological, digital, pain, or multidisciplinary input when fear interferes with recovery. The triggers and progress signals are illustrative and should be adapted to validated local instruments, medical stability, baseline function, comorbidity, available services, and patient goals. The pathway requires prospective validation before it can be treated as a standard of care.

**Table 3:** Proposed stepped-care pathway.

Level	Illustrative trigger	Core actions	Illustrative progress signal
Step 1: Universal TKA care	All patients	Evidence-based standard rehabilitation: realistic education, multimodal analgesia, supported mobilization, progressive exercise, teach-back, written/digital plan	Medical stability; understands expected symptoms/red flags; begins functional progression
Step 2: Enhanced fear-informed rehabilitation	Observed avoidance affecting milestones, elevated fear on a validated local measure, high catastrophizing, low confidence, or stalled task progression	Identify feared tasks; collaborative hierarchy; closer supervision; behavioral experiments; feedback/mastery; family coaching; repeat multidomain assessment	Fear/avoidance decreases or patient-valued task performance progresses over locally appropriate follow-up
Step 3: Targeted psychological or technology-supported adjunct	Persistent fear/distress or repeated avoidance despite Step 2; rehabilitation plateau without an untreated medical cause	CBT-informed intervention within scope; psychology/pain referral when indicated; selected motor imagery, VR, mobile/digital support, relaxation, or telerehabilitation monitoring	Improved participation, adherence, confidence, function, or pain interference; plan revised if no meaningful response
Step 4: Multidisciplinary reassessment	Worsening pain/fear, red flags, marked functional loss, new neurological/hemodynamic concerns, or failure to progress	Orthopedic/medical assessment; pain phenotype; imaging/laboratory work-up as indicated; medication review; rehabilitation and psychological reassessment	Correctable causes addressed and a revised shared plan established

This framwork has not been prospectively validated as a clinical algorithm. Triggers and progress signals require local adaptation and should not replace medical assessment or individualized clinical judgment.

## 8 Implementation and audit measures

Implementation requires coordination across orthopedics, anesthesia/pain services, nursing, physical therapy, occupational therapy, primary care, and psychology. A common communication approach can define expected symptoms, safe loading, red flags, and progression while avoiding contradictory reassurance or threat-amplifying language. Electronic documentation can prompt assessment, record observed avoidance and relevant TSK items, document a fear hierarchy or functional goal, and trigger referral when appropriate.

Table 4 presents illustrative local audit measures rather than externally validated quality standards. No universal numerical performance targets are specified because evidence-based benchmarks for these measures were not identified. Each service should establish a local baseline, define an achievable improvement target, report numerator/denominator data where appropriate, and monitor balancing measures such as falls, wound events, readmissions, and unplanned contacts.

**Table 4:** Illustrative implementation and audit measures. No numerical target is presented as evidence-based.

Measure	Operational definition	Suggested reporting approach
Fear assessment coverage	Patients with documented preoperative and early postoperative assessment of movement-related fear when locally feasible	Report numerator/denominator and stratify by setting; establish a local baseline and improvement goal rather than a universal percentage.
First mobilization process	Eligible patients completing planned supported mobilization, with reasons for delay documented	Report timing distribution and documented causes of delay; compare with the local ERAS/rehabilitation pathway.
Enhanced care-plan documentation	Patients with clinically important fear/avoidance who have a documented functional goal, fear-informed strategy, and follow-up plan	Report coverage and reasons for noncompletion; use local improvement cycles.
Reassessment	Patients receiving enhanced care who have repeat fear/TSK and functional assessment within the locally defined follow-up window	Report follow-up completion and change distributions; avoid assuming a universal clinically important TSK change.
Functional recovery	Change in sit-to-stand, walking, stairs, range of motion/strength, and patient-specific activity as appropriate	Report distributions, responder definitions where validated, and missing data.
Patient-reported recovery	Change in kinesiophobia/fear plus pain interference, confidence/self-efficacy, adherence, and participation	Report the TSK version/language and descriptive change; interpret alongside function.
Safety and unplanned care	Falls, wound events, readmissions, urgent contacts, adverse exercise events, or delayed diagnosis	Use as balancing measures and compare with the service baseline; investigate any adverse trend.

Services should establish local baselines and improvement goals and monitor balancing measures.

## 9 Discussion

### 9.1 Principal findings

The central finding of this review is that kinesiophobia after TKA is clinically relevant and potentially modifiable, but the direct intervention evidence is narrower than the broader rehabilitation literature. Direct TKA studies support the plausibility of multicomponent fear-focused functional exercise, structured education, targeted CBT-informed approaches, and selected digital, VR, imagery, or motivational adjuncts. However, many trials are small, single-center, short-term, or heterogeneous in intervention content and TSK measurement. These limitations preclude formal certainty-based recommendation grading and require cautious, intervention-specific interpretation.

Several components that remain important to patient care—multimodal analgesia, early supported mobilization, strengthening, gait training, and telerehabilitation—are supported by general TKA rehabilitation evidence and current guidance (28, 29, 44). Their role in a kinesiophobia pathway is best understood as providing the physical and clinical conditions in which feared movement can be practiced safely. They should not be cited as direct proof of kinesiophobia prevention or treatment unless fear outcomes were measured.

Measurement remains a major source of uncertainty. TSK versions, thresholds, language adaptations, time points, and outcome selection vary. A statistically lower TSK score does not automatically represent a clinically important change, and reduced fear without improved activity or participation may have limited patient-level meaning. Future studies should report the TSK version, validated language, threshold rationale, baseline distribution, measurement error or meaningful-change assumptions, and parallel functional outcomes.

### 9.2 Clinical controversies and evidence boundaries

Universal versus targeted psychological intervention remains unresolved. Brief education and supportive communication can be integrated broadly, whereas formal CBT is resource intensive and has inconsistent effects on pain and knee function; targeted use for persistent high fear or distress is more defensible than universal intensive therapy. Whether fear reduction is an intermediate outcome or a sufficient treatment target also remains uncertain: trials should determine whether change in kinesiophobia translates into adherence, function, participation, and valued activities.

The role of graded exposure requires similar precision. Direct TKA evidence supports fear-focused functional exercise as part of a multicomponent intervention (33), but robust trials isolating graded exposure from exercise, education, therapist attention, and self-efficacy support are lacking. The proposed use of a fear hierarchy should therefore be viewed as an evidence-informed clinical technique rather than a validated TKA-specific protocol.

Passive adjuncts may provide short-term symptom relief, but their mechanism and durability are uncertain and they may inadvertently reinforce dependency if presented as necessary for movement. Early mobilization also requires individualized judgment: delay caused by fear should be addressed, but physiological instability, uncontrolled pain, or a surgical complication must not be reframed as avoidance.

### 9.3 Strengths and limitations

Strengths of this review include explicit separation of direct fear-specific evidence from broader TKA rehabilitation evidence, integration of rehabilitation, nursing, psychological, perioperative, and technology-supported perspectives, attention to TSK measurement limitations, and translation of the evidence into clinically interpretable statements. The screening schedule, stepped-care pathway, and audit measures are deliberately presented as proposed implementation frameworks rather than validated algorithms or universal standards.

Important limitations remain. The review is narrative rather than systematic; searches were not independently duplicated; Embase, CINAHL, PsycINFO, Scopus, and Web of Science were not independently searched; and no *de novo* study-level risk-of-bias table or formal certainty assessment was performed. Although recent multidisciplinary systematic reviews were used to broaden disciplinary coverage, relevant studies may have been missed. Several direct trials are small, single-center, or short-term; intervention content and co-interventions vary; and publication bias cannot be excluded. Evidence published in 2025-2026 has had limited opportunity for replication. The proposed clinical pathway should therefore be prospectively tested and updated as larger multicenter trials emerge.

### 9.4 Research priorities

- Establish TKA- and language-specific interpretability data for TSK-17, TSK-13, and TSK-11, including measurement error and clinically important change across perioperative time points.
- Develop and externally validate pragmatic risk models that integrate fear, catastrophizing, self-efficacy, pain phenotype, physical capacity, and social context without treating psychological scores as deterministic labels.
- Test fear-focused functional exercise and graded exposure using adequately powered multicenter designs that isolate intervention components, dose, fidelity, and therapist training.
- Compare universal brief education with risk-stratified CBT-informed or psychologically informed rehabilitation using outcomes that include activity, adherence, participation, and adverse events in addition to TSK scores.
- Determine which patients benefit from VR, motor imagery, mobile health, motivational procedures, or telerehabilitation, and report accessibility, digital inequity, implementation burden, and cost-effectiveness.
- Evaluate the proposed screening schedule, stepped-care pathway, and audit measures prospectively before adopting fixed thresholds or performance targets.

## 10 Conclusion

Kinesiophobia should be considered a potentially modifiable barrier within the TKA rehabilitation pathway, but the strength and directness of evidence differ across interventions. Direct TKA evidence is most persuasive for selected multicomponent fear-focused, educational, cognitive-behavioral, and technology-supported interventions, while analgesia, early mobilization, progressive exercise, and telerehabilitation are primarily supported as components of general TKA care. TSK scores should be interpreted in a multidomain context, with no universal assumption that a TSK-17 threshold around 37 or any TSK-11 threshold applies across languages and recovery phases. The screening schedule, stepped-care pathway, and audit measures proposed here are intended for local adaptation and prospective evaluation rather than use as validated algorithms or evidence-derived standards.

## References

[1] Steinmetz JD Culbreth GT Haile LM Rafferty Q Lo J Fukutaki KG. Global, regional, and national burden of osteoarthritis, 1990–2020 and projections to 2050: a systematic analysis for the global burden of disease study 2021. Lancet Rheumatol. (2023) 5:e508–22. 10.1016/S2665-9913(23)00163-7 37675071 PMC10477960

[2] DeFrance MJ Scuderi GR. Are 20% of patients actually dissatisfied following total knee arthroplasty? A systematic review of the literature. J Arthroplasty. (2023) 38:594–9. 10.1016/j.arth.2022.10.011 36252743

[3] Price AJ Alvand A Troelsen A Katz JN Hooper G Gray A. Knee replacement. Lancet. (2018) 392:1672–82. 10.1016/S0140-6736(18)32344-4 30496082

[4] Du X Shao Y Xue J Kong J. Prevalence and influencing factors of kinesiophobia after total knee arthroplasty: a systematic review and meta-analysis. J Orthop Surg Res. (2025) 20:332. 10.1186/s13018-025-05752-w 40170179 PMC11959722

[5] Brown OS Hu L Demetriou C Smith TO Hing CB. The effects of kinesiophobia on outcome following total knee replacement: a systematic review. Arch Orthop Trauma Surg. (2020) 140:2057–70. 10.1007/s00402-020-03582-5 32839826

[6] Bakırhan S Unver B Elibol N Karatosun V. Fear of movement and other associated factors in older patients with total knee arthroplasty. Ir J Med Sci. (2023) 192:2217–22. 10.1007/s11845-022-03214-4 36445627

[7] Yan Z Wu Y Liu M Wang X Wang J Wang Z. Heterogeneous trajectories of kinesiophobia and their effects on rehabilitation outcomes after total knee arthroplasty: a prospective cohort study. J Orthop Surg Res. (2023) 18:449. 10.1186/s13018-023-03881-8 37353854 PMC10290332

[8] Zhou Y Gao W Gao S Guo X Liu M Cao C. Pain catastrophizing, kinesiophobia and exercise adherence in patients after total knee arthroplasty: the mediating role of exercise self-efficacy. J Pain Res. (2023) 16:3993–4004. 10.2147/JPR.S432106 38026453 PMC10676101

[9] Zhou Y Gao W Cao Z Gao S Guo X Liu M. Network analysis of pain catastrophizing, self-efficacy, and kinesiophobia among patients after total knee arthroplasty: a cross-sectional study. Patient Prefer Adherence. (2024) 18:1897–906. 10.2147/PPA.S452773 39310086 PMC11414755

[10] Yao Y Zhang Q Cui S Guo X. Study on the impact of kinesiophobia after total knee arthroplasty on patients during hospitalization: a pilot study. PLoS One. (2025) 20:e0317774. 10.1371/journal.pone.0317774 39879177 PMC11778713

[11] Xing D Chen Y Qin Y Zhang Y Li Y Jia T. Analysis of factors influencing postoperative kinesiophobia in elderly total knee arthroplasty patients based on health ecology theory: a cross-sectional study. Medicine (Baltimore). (2025) 104:e45660. 10.1097/MD.0000000000045660 41261577 PMC12582761

[12] Qu W Yan Z Wei H Zhang S Wang X Liu M. Heterogeneity of social participation in patients three months after total knee arthroplasty: a latent profile analysis. BMC Musculoskelet Disord. (2024) 25::619. 10.1186/s12891-024-07744-z 39090646 PMC11295653

[13] Yılmaz GG Akel BS Saitoğlu Y Aki E. Occupational self-perception level effects on the development of kinesiophobia in individuals with total knee arthroplasty. J Orthop. (2023) 42:80–6. 10.1016/j.jor.2023.07.020 PMC10382608 37519913

[14] Kuwahara K Kato T Akatsuka Y Nakazora S Fukuda A Asada K. Development of a clinical prediction rule for mobility status at discharge in patients with total knee arthroplasty: using a decision tree model. J Orthop Sci. (2024) 29:1451–5. 10.1016/j.jos.2023.12.002 38114367

[15] Vogel M Binneböse M Lohmann CH Junne F Berth A Riediger C. Are anxiety and depression taking sides with knee-pain in osteoarthritis? J Clin Med. (2022) 11:1094. 10.3390/jcm11041094 35207366 PMC8876729

[16] Springborg AH Visby L Kehlet H Foss NB. Psychological predictors of acute postoperative pain after total knee and hip arthroplasty: a systematic review. Acta Anaesthesiol Scand. (2023) 67:1322–37. 10.1111/aas.14301 37400963

[17] Hung TH Liu MC Kuo YW Lee JD. Comparative analysis of multidisciplinary complementary intervention versus routine physiotherapy rehabilitation to reduce kinesiophobia in patients with knee osteoarthritis undergoing total knee replacement: a systematic review and meta-analysis. Sports Med Open. (2025) 11:91. 10.1186/s40798-025-00899-6 40770481 PMC12328877

[18] Dupuis F Cherif A Batcho C Massé-Alarie H Roy JS. The Tampa scale of kinesiophobia: a systematic review of its psychometric properties in people with musculoskeletal pain. Clin J Pain. (2023) 39:236–47. 10.1097/AJP.0000000000001104 36917768

[19] Alpalhão V Vaz JR Cordeiro N De P Correia P. Are the cross-culturally adapted versions of the Tampa scale for kinesiophobia 11-item valid, reliable, and responsive? A COSMIN-informed systematic review of measurement properties. J Pain. (2024) 25:104602. 10.1016/j.jpain.2024.104602 38866123

[20] Najafi F Zare Z Mortazavi SM Lundberg M Shahsavari H. Overcoming fear of movement resulting from knee replacement; strategies used by patients: an interview study. Int J Orthop Trauma Nurs. (2022) 45:100904. 10.1016/j.ijotn.2021.100904 35181253

[21] Lee J Kim M Lee Y. Motor imagery enhances early rehabilitation in patients with high kinesiophobia after total knee arthroplasty: a randomized controlled trial. J Rehabil Med. (2025) 57:jrm43688. 10.2340/jrm.v57.43688 41327758 PMC12676586

[22] Paredes AC Costa P Almeida A Pinto PR. Presurgical anxiety and acute postsurgical pain predict worse chronic pain profiles after total knee/hip arthroplasty. Arch Orthop Trauma Surg. (2025) 145:118. 10.1007/s00402-024-05681-z 39798042

[23] Reis N Altinbas B. Nurse-led preoperative education on kinesiophobia in older adult patients undergoing total knee arthroplasty: a randomized controlled trial. Geriatr Nurs. (2026) 71:104124. 10.1016/j.gerinurse.2026.104124 42258910

[24] Song L-X Yang L Li Y Lei F-Q Qin Y Wang L-H. Influence of health education based on the transtheoretical model on kinesiophobia levels and rehabilitation outcomes in elderly patients undergoing total knee arthroplasty. Heliyon. (2024) 10:e32445. 10.1016/j.heliyon.2024.e32445 38975135 PMC11225756

[25] İmrek AK Yilmaz M. The effect of preoperative education on fear of first mobilization experienced after total knee arthroplasty in individuals. Int J Orthop Trauma Nurs. (2025) 57:101189. 10.1016/j.ijotn.2025.101189 40367643

[26] Nguyen C Boutron I Roren A Anract P Beaudreuil J Biau D. Effect of prehabilitation before total knee replacement for knee osteoarthritis on functional outcomes: a randomized clinical trial. JAMA Netw Open. (2022) 5:e221462. 10.1001/jamanetworkopen.2022.1462 35262716 PMC8908069

[27] Tazreean R Nelson G Twomey R. Early mobilization in enhanced recovery after surgery pathways: current evidence and recent advancements. J Comp Eff Res. (2022) 11:121–9. 10.2217/cer-2021-0258 35045757

[28] Wainwright TW Gill M McDonald DA Middleton RG Reed M Sahota O. Consensus statement for perioperative care in total hip replacement and total knee replacement surgery: enhanced recovery after surgery (ERAS) society recommendations. Acta Orthop. (2020) 91:3–19. 10.1080/17453674.2019.1683790 31663402 PMC7006728

[29] Bove AM Carroll LA Cone S Dibblee P Hensley CP Lenington K. Clinical practice guideline for physical therapist management of total knee arthroplasty: revision 2026. Phys Ther. (2026) 106:pzag058. 10.1093/ptj/pzag058 42506877 PMC13403188

[30] Changjun C Jingkun L Yun Y Yingguang W Yanjun R Debo Z. Enhanced recovery after total joint arthroplasty (TJA): a contemporary systematic review of clinical outcomes and usage of key elements. Orthop Surg. (2023) 15:1228–40. 10.1111/os.13710 36971112 PMC10157715

[31] Jette DU Hunter SJ Burkett L Langham B Logerstedt DS Piuzzi NS. Physical therapist management of total knee arthroplasty. Phys Ther. (2020) 100:1603–31. 10.1093/ptj/pzaa099 32542403 PMC7462050

[32] Doyğacı AG Guler S Esen E. Effectiveness of a first mobilization protocol in patients undergoing knee arthroplasty: a randomized contolled trial. J Perianesth Nurs. (2026) 41:1150-63. 10.1016/j.jopan.2026.05.002 42455081

[33] Monticone M Ferrante S Rocca B Salvaderi S Fiorentini R Restelli M. Home-based functional exercises aimed at managing kinesiophobia contribute to improving disability and quality of life of patients undergoing total knee arthroplasty: a randomized controlled trial. Arch Phys Med Rehabil. (2013) 94:231–9. 10.1016/j.apmr.2012.10.003 23063624

[34] Çetinkaya F Karakoyun A. The effects of elastic band exercise on the pain, kinesiophobia, functional, and psychological status after total knee arthroplasty: a randomized controlled trial. Clin Rheumatol. (2022) 41:3179–88. 10.1007/s10067-022-06266-0 35776283

[35] Bordeleau M Vincenot M Lefevre S Duport A Seggio L Breton T. Treatments for kinesiophobia in people with chronic pain: a scoping review. Front Behav Neurosci. (2022) 16:933483. 10.3389/fnbeh.2022.933483 36204486 PMC9531655

[36] Liu K Liu Y Ma X Fu D Fan Z. Effect of cognitive behavioral therapy on pain, knee function, and psychological status in patients after primary total knee arthroplasty: a systematic review and meta-analysis. BMC Musculoskelet Disord. (2024) 25:280. 10.1186/s12891-024-07413-1 38605391 PMC11007994

[37] Kong Y An S Zhang W Wu S An S. Potential role of peri-operative cognitive behavioral therapy on pain and joint function after total knee arthroplasty: a meta-analysis. Indian J Orthop. (2026) 60:18–29. 10.1007/s43465-025-01553-9 41541013 PMC12799857

[38] Cai L Gao H Xu H Wang Y Lyu P Liu Y. Does a program based on cognitive behavioral therapy affect kinesiophobia in patients following total knee arthroplasty? A randomized, controlled trial with a 6-month follow-up. J Arthroplasty. (2018) 33:704–10. 10.1016/j.arth.2017.10.035 29239772

[39] Sun JN Chen W Zhang Y Zhang Y Feng S Chen XY. Does cognitive behavioral education reduce pain and improve joint function in patients after total knee arthroplasty? A randomized controlled trial. Int Orthop. (2020) 44:2027–35. 10.1007/s00264-020-04767-8 32772319

[40] Chen W Sun JN Hu ZH Zhang Y Chen XY Feng S. Cognitive behavioral therapy cannot relieve postoperative pain and improve joint function after total knee arthroplasty in patients aged 70 years and older. Aging Clin Exp Res. (2021) 33:3293–302. 10.1007/s40520-021-01870-7 33991330

[41] Buvanendran A Sremac AC Merriman PA Valle D Burns CJ McCarthy JW. Preoperative cognitive-behavioral therapy for reducing pain catastrophizing and improving pain outcomes after total knee replacement: a randomized clinical trial. Reg Anesth Pain Med. (2021) 46:313–21. 10.1136/rapm-2020-102258 33452201

[42] Zhu S Xu Y Wang L Chen J Luo A. Efficacy of cognitive behavioral therapy for kinesiophobia: a systematic review and meta-analysis of randomized controlled trials. J Pain Res. (2025) 18:3919–32. 10.2147/JPR.S526179 40791870 PMC12336382

[43] Li N Yao X Ji H. Relationships among disease knowledge, social support, anxiety and self-efficacy in patients after total knee arthroplasty: a chain mediating effect. Nurs Open. (2023) 10:4728–36. 10.1002/nop2.1723 36879531 PMC10277396

[44] Konnyu KJ Thoma LM Cao W Aaron RK Panagiotou OA Bhuma MR. Rehabilitation for total knee arthroplasty: a systematic review. Am J Phys Med Rehabil. (2023) 102:19–33. 10.1097/PHM.0000000000002008 35302953 PMC9464796

[45] Lu G Wu T Tan Q Wu Z Shi L Zhong Y. The effect of a micro-visual intervention on the accelerated recovery of patients with kinesiophobia after total knee replacement during neo-coronary pneumonia. Medicine (Baltimore). (2021) 100:e24141. 10.1097/MD.0000000000024141 33578519 PMC7886397

[46] Kara A Karabulut N. The effect of mobile-based training on anxiety, kinesiophobia, and physical function in patients undergoing total knee arthroplasty. J Arthroplasty. (2026) S0883-5403:00701-1. 10.1016/j.arth.2026.06.075 42373024

[47] Alptekin HM Ulubay S. The effect of an artificial intelligence-assisted motivational procedure on kinesiophobia and mobility after total knee arthroplasty: a randomized controlled trial. BMC Musculoskelet Disord. (2026) 27:465. 10.1186/s12891-026-09849-z 42010557 PMC13224616

[48] Gür O Başar S. The effect of virtual reality on pain, kinesiophobia and function in total knee arthroplasty patients: a randomized controlled trial. Knee. (2023) 45:187–97. 10.1016/j.knee.2023.09.012 37931366

[49] Menteş Z Çetinkaya F. Effect of virtual reality-based relaxation exercise on postoperative pain and kinesiophobia. Pain Manag Nurs. (2026) S1524-9042:00127-X. 10.1016/j.pmn.2026.03.017 41991400

[50] Su S He J Wang R Chen Z Zhou F. The effectiveness of virtual reality, augmented reality, and mixed reality rehabilitation in total knee arthroplasty: a systematic review and meta-analysis. J Arthroplasty. (2024) 39:582–590.e4. 10.1016/j.arth.2023.08.051 37598785

[51] Song S Liu Z Zhang Q. Application of virtual reality technology in postoperative rehabilitation following total knee arthroplasty: a scoping review. Int J Orthop Trauma Nurs. (2024) 54:101124. 10.1016/j.ijotn.2024.101124 39096627

[52] Gazendam A Zhu M Chang Y Phillips S Bhandari M. Virtual reality rehabilitation following total knee arthroplasty: a systematic review and meta-analysis of randomized controlled trials. Knee Surg Sports Traumatol Arthrosc. (2022) 30:2548–55. 10.1007/s00167-022-06910-x 35182172 PMC8857886

[53] Tsang MP Man GCW Xin H Chong YC Ong MTY Yung PSH. The effectiveness of telerehabilitation in patients after total knee replacement: a systematic review and meta-analysis of randomized controlled trials. J Telemed Telecare. (2024) 30:795–808. 10.1177/1357633X221097469 35549756

[54] Özbaş N Şahan N Aydın E. The effect of foot reflexology on pain and kinesiophobia in patients following total knee arthroplasty: a double-blind randomized controlled study. Orthop Nurs. (2024) 43:223–33. 10.1097/NOR.0000000000001043 39047275

[55] Pritwani S Shrivastava P Pandey S Kumar A Malhotra R Maddison R. Mobile and computer-based applications for rehabilitation monitoring and self-management after knee arthroplasty: scoping review. JMIR Mhealth Uhealth. (2024) 12:e47843. 10.2196/47843 38277195 PMC10858429

